# Can algae contribute to the war with Covid-19?

**DOI:** 10.1080/21655979.2021.1910432

**Published:** 2021-04-15

**Authors:** Wen Yi Chia, Hanz Kok, Kit Wayne Chew, Sze Shin Low, Pau Loke Show

**Affiliations:** a Department of Chemical and Environmental Engineering, Faculty of Science and Engineering, University of Nottingham Malaysia, Semenyih, Selangor Darul Ehsan, Malaysia; b School of Energy and Chemical Engineering, Xiamen University Malaysia, Sepang, Selangor, Malaysia; c College of Chemistry and Chemical Engineering, Xiamen University, Xiamen, Fujian, China; d Biosensor National Special Laboratory, Key Laboratory for Biomedical Engineering of Education Ministry, Department of Biomedical Engineering, Zhejiang University, Hangzhou, PR China

**Keywords:** Antiviral compounds, microalgal extracts, sars-cov-2, vaccines, dietary supplement

## Abstract

The world at large is facing a new threat with the emergence of the Coronavirus Disease 2019 (COVID-19) pandemic. Though imperceptible by the naked eye, the medical, sociological and economical implications caused by this newly discovered virus have been and will continue to be a great impediment to our lives. This health threat has already caused over two million deaths worldwide in the span of a year and its mortality rate is projected to continue rising. In this review, the potential of algae in combating the spread of COVID-19 is investigated since algal compounds have been tested against viruses and algal anti-inflammatory compounds have the potential to treat the severe symptoms of COVID-19. The possible utilization of algae in producing value-added products such as serological test kits, vaccines, and supplements that would either mitigate or hinder the continued health risks caused by the virus is prominent. Many of the characteristics in algae can provide insights on the development of microalgae to fight against SARS-CoV-2 or other viruses and contribute in manufacturing various green and high-value products.

## Graphical Abstract

**Figure:**
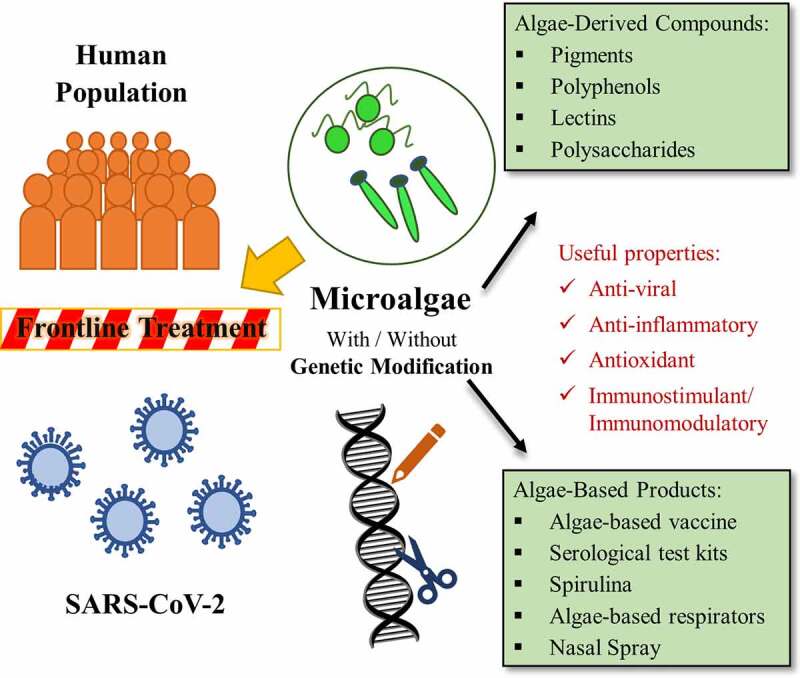

## Introduction

The novel corona virus (2019-nCoV), more commonly identified as the coronavirus disease 2019 (COVID-19), named officially by the World Health Organization (WHO) on the 12^th^ of January 2020, had its first outbreak in the Huanan South China Seafood Market, located in Wuhan City, Hubei Province, China [1]. It was first made known by a continuous diagnosis of an unknown acute respiratory tract infection. Upon further testing, the unknown virus at the time was found to be identified as a coronavirus that was largely similar with the bat coronavirus and the SARS-CoV [2]. This newly identified β-coronavirus is known as the SARS-CoV-2, an enveloped non-segmented positive-sense RNA virus, which is included in *sarbecovirus, Orthocoronavirinae* subfamily [3].

The number of COVID-19 cases have been increasing exponentially, with the virus spreading rapidly to all parts of the world, causing the WHO to declare the COVID-19 outbreak as a pandemic on the 11^th^ of March 2020 [4]. As of March 1, 2021, more than one hundred million cases of COVID-19 with a death toll of more than two million have been reported. The main source of transmission of SARS-CoV-2 was suspected to be the direct contact of intermediate host animals or the consumption of wild animals. Even though COVID-19 cases had a significant outbreak in China, clusters of people in other countries that had no history of travel to or from China was found to be infected. Matters got worse as the infection was found to be transmitted from people before any onset of symptoms could be found as well as those who were asymptomatic [5]. The usual symptoms of COVID-19 would include common ailments such as fever, cough, sore throat, headache, breathlessness, and fatigue [6]. However, some of these symptoms were not found in certain patients signifying that the virus has an asymptomatic clinical feature as well. As a patient’s condition worsens, the patient may suffer from severe pneumonia, respiratory tract infection, septic shock and progressive organ failure ultimately leading to death, as experienced by a great number of COVID-19 victims. People groups who are most vulnerable to the virus are the elderly, young children, pregnant women and people with preexisting medical conditions such as diabetes, cardiovascular problems, hypertension etc, along with people who are immuno-compromised including patients with human immunodeficiency virus (HIV), cancer and auto-immune disorders [2]. Most patients who develop critical clinical symptoms and eventually lead to death are those with preexisting medical conditions.

A mitigation aspect for the pandemic that should be discussed in length is to find specific clinical treatments to fight the disease. Current front-line treatment needs consist of corticosteroid and antivirals therapy, along with mechanical respiratory support [7]. In the case of vaccines, even though some biotechnological companies have developed vaccines for the virus, it would likely take a few more years before there is global access to it [8]. Therefore, it is urgent to study and develop anti-inflammatory drugs, antibodies, and antiviral drugs as short-term treatment methods to those who have been infected, whilst placing priority in accelerating the production of vaccines that would help in the long run [9].

A more conventional method of forming these products includes the usage of plants as they can be used to produce bioactive metabolites and biopharmaceuticals [9]. However, the use of microalgae may be favored over plants as a result of their lower production costs, high scalability, and increased biomass culture with simple mineral requirements [9]. Algae cultures in general are also more advantageous in the biopharmaceutical production as there is not much of a need to compete for agricultural land that is increasing in scarcity [10]. This makes the cultivation of microalgae a sustainable and green approach, being incredible cellular factories for the production of compounds with high value [11]. Since microalgal metabolism changes its intracellular environment in response to the changes in the external environment, biosynthesis of specific compound can be stimulated by manipulating the metabolic pathways including culture conditions to improve photosynthetic growth [12,13]. Their special metabolic patterns are closely linked to these unique features of the environments which produce an extraordinary of active secondary metabolites that are often unique and different from those identified in terrestrial organisms [14]. Therefore, there is a large prospect in the exploitation of microalgae for further development into pharmaceutical products, allowing them to produce products that are valuable commercially such as proteins, polysaccharides, phycobiliproteins, polyunsaturated fatty acids, and carotenoids, either sourced directly from primary metabolism or synthesized from secondary metabolism [15,16].

According to Sami et al. [18], algae can be recommended as a treatment to SARS-Cov-2, both preventive and curative. In fact, algae and cyanobacteria can be referred to as one of the richest manufacturers of bioactive metabolites which are pharmacologically active and exhibit antiviral properties [17]. For instance, antiviral efficacy of algal polysaccharides including carrageenan, fucoidan, ulvan, agar, and alginates have been investigated against various viruses such as dengue virus (DENV) and HIV, besides being natural polymers which are low cost, biodegradable, non-toxic, and biocompatible [18]. It was suggested that sulfated polysaccharides can inhibit viral infection by interfering with S-protein of SARS-CoV-2 that binds to the heparan sulfate co-receptor in host tissues as Kwon et al. [19] reported in vitro results of tight binding between specific sulfated polysaccharides and the S-protein. The application of these non-anticoagulant polysaccharides could be oral delivery, metered dose inhaler or nasal spray, since heparin is not orally bioavailable but fucoidans extracted from edible sulfated seaweed polysaccharides are in the category of Generally Recognized as Safe (GRAS) [19].

On the other hand, C-phycocyanin, a pigment-binding protein found in *Spirulina* blue-green algae, enhances anti-tumor, anti-inflammation, and antioxidation activities [20]. Tzachor et al. [21] used aqueous extracts of *Spirulina* as a therapy for cytokine storm and reported a reduction of tumor necrosis factor (TNF)-α secretion levels induced by macrophage and monocyte. Anti-TNF therapy or TNF-α blockers are important to reduce inflammation-driven capillary leak caused by the key inflammatory cytokines which deteriorates the lung function of COVID-19 patients [22]. As a novel algae-based bioactive compounds for anti-TNF treatment, *Spirulina* has an added advantage since it is approved by Food Drug Administration (FDA) as a dietary supplement with non-intrusive administration and orally absorbing property [21]. With proven therapeutic potential of various algal compounds, this short review discusses the potential utilization of micro/macroalgae and cyanobacteria in producing high value products, which could help in combating Covid-19.

## Potential of microalgal value-added products

Natural sources need to be explored to produce new pharmaceutical approaches against SARS-CoV-2. Algae and cyanobacteria with a chemo-diversity can be identified as a relevant origin for the development of antiviral therapy since they possess both virus suppressing properties and immunity improving capacity [18]. Table 1 shows different types of algae-derived compounds with different therapeutic properties. It is noteworthy that algal-derived lectins and polysaccharides could serve as potential antiviral agents. Algal polysaccharides such as carrageenan, fucans, nostoflan, naviculan, alginates, calcium spirulan, galactans, and laminarin have been tested in term of their antiviral efficacy toward viruses such as DENV, HIV, hepatitis A virus (Hep A) virus and herpes simplex virus-1 and −2 (HSV-1 and HSV-2). Lectins, such as cyanovirin, microvirin, griffithsin, scytovirin, and phlorotannins, which are proteins that bind reversibly to certain mono and oligosaccharides that are lacking catalytic activity, have also been tested to counteract several viruses including HIV [18]. Another promising agent is a polypeptide derived from microalgae called griffithsin (GRFT). GRFT has demonstrated the inhibition of viral entry for the cases of the HIV, the SARS-CoV-1 and the MERS-CoV along with other algae-derived lectins such as cyanovirin-N (CVN), isolated from the *Nostoc ellipsosporum* cyanobacteria, scytovirin (SVN) from *Scytonema varium* and agglutinin from *Oscillatoria agardhii* [9].

**Table 1.** A comparison of different algae-derived compounds [9,18]

Algae-Derived Antiviral Compound	Sub-category of Compound	Properties
Polysaccharides	Carrageenan, a sulfated polysaccharide found in Rhodophyta such as *Chondrus, Gigartina, Hypnea*, and *Eucheuma*	Low molecular weight ι-carrageenan Penetrate host cell and inhibits viral replication Block attachment of Herpes Simplex Virus (HSV) and Dengue Virus Effective against human papillomavirus (HPV) Exhibit considerable inhibitory effects against influenza virus Nasal spray product reduced symptoms of common cold, viral load and inflammation, active against Influenza A virus with zanamivir addition
	Fucan, a high molecular weight sulfated polysaccharide found in brown algae	Fucoidan Anti-HIV activity Strong inhibitory effect on the reverse transcriptase enzyme of HIV-1 Anti-influenza A virus (IAV) Anti-HSV Improves immune function
	Naviculan, isolated from *Navicula directa*	Anti-HSV-1, HSV-2 and influenza A virus
	Ulvan, a sulfated polysaccharide from Chlorophyta	Anti-HSV-1 Antiviral activity against Japanese encephalitis virus
	Calcium spirulan from *Arthrospira platensis*	Inhibits viral replication of HSV-1, mumps virus, measles virus, HIV-1 and IAV by blocking the virus before penetrating host cells
	Nostoflan from *Nostoc flagelliforme*	Show antiviral activity against viruses which have carbohydrates as cellular receptors Inhibits viral replication of influenza A virus, HSV-1, HSV-2, and human cytomegalovirus
	Alginates, derived from brown algae like *Ascophyllum nodosum, Laminaria digitata, Laminaria japonica, Laminaria hyperborean* and *Macrocystis pyrifera*	Significantly inhibit the acute infection of MT4 cells and the chronic infection of H9 cells with HIV-1 Sulfated form of alginate i.e. sulfated polymannuroguluronate (SPMG) inhibits HIV-1 infection
	Galactans, from red algae *Agardhiella tenera, Schizymenia binderi*, Brazilian marine alga *Cryptonemia crenulata* and marine alga *Callophyllis variegate*	Exhibit antiviral potency against enveloped viruses including HSV-1 and HSV-2, DENV, HIV-1 and HIV-2, and hepatitis A virus (Hep A) virus
	Laminaran, from brown seaweeds like *Ecklonia kurome, Eisenia bicyclis, Laminaria japonica*	Great antiviral activity and are bio-compatible Prevent adsorption of HIV reverse transcriptase
Lectins	Griffithsin from *Griffithsia* sp.	Targets high-mannose arrays at the surface of pathogenic enveloped viruses like HIV, SARS-CoV-1 and MERS-CoV Binds to the SARS-CoV-1 spike (S) glycoprotein, inhibiting viral entry, accompanied with a specific inhibition of deleterious host immune reactions in response to SARS Does not exhibit cytotoxicity
	Cyanovirin-N (CVN) from *Nostoc ellipsosporum*	Inhibit viral entry for HIV, Ebola, simian immunodeficiency virus (SIV), feline immunodeficiency virus and influenza virus
	Scytovirin (SVN) from *Scytonema varium*	Antiviral against Marburg virus, Zaire ebolavirus, HIV, and SARS-CoV
	Agglutinin from *Oscillatoria agardhii*	Anti-HIV activity
Pigments	Phycobilins from cyanobacteria like *Oscillatoria* sp., *Nostoc* sp., *Anabaena* sp. and *Spirulina* sp.	Antioxidant and anti-inflammatory Photosynthetic properties
	Phycoerythrin from Rhodophyta and cyanobacteria	Antitumor Anti-aging Anti-inflammatory
	Fucoxanthin, a xanthophyll-like carotenoid	Anti-inflammatory
	Zeaxanthin and lutein from *Chlorella protothecoides, D.salina*, and *Spirulina maxima*	Anti-inflammatory against endotoxin-induced uveitis
	Violaxanthin, natural xanthophyll from *Chlorella ellipsoidea* and *Dunaliella tertiolecta*	Anti-inflammatory
Polyphenols	Phlorotannins from brown algae	Anti-allergic Antioxidant Photoprotective Substantial bioactivity in the Influenza virus, HIV, and Hepatitis Virus
	Diterpenes from *Dictyota pfaffi and Dictyota menstrualis*	Anti-HIV activity with low toxicity
	Fucosetrol from brown algae such as *Eisenia bicyclis, Fucus vesiculosus and Turbinaria conoides*	In vitro properties Efficient therapeutic agent
	Other polyphenolic compounds that are algal species specific	Antioxidant, antimicrobial, anti-cancer, anti-inflammatory, antiallergic, antidiabetic, anti-aging

Other than antiviral agents, algal nutraceuticals with their anti-inflammatory, antimicrobial, immunostimulatory, and immunomodulatory properties are also important for boosting immunity, preventing diseases and treating disorders associated with the severe SARS-CoV-2 infections, such as anti-inflammatory treatment and tissue repair [23]. For instance, C-phycoyanin, a pigment-binding protein, with the properties of anti-inflammation, antioxidation, and anti-tumor was tested to reduce the secretion levels of a protein, which causes cytokine storms in COVID-19 patients [21]. Although it will not replace SARS-CoV-2 vaccinations, the algae extract may be used a dietary supplement to prevent cytokine storms once the patients are diagnosed, especially those high-risk populations including the elderly and those with severe medical conditions [24]. This is because the influx of those pro-inflammatory cytokines such as TNF-α, interleukin (IL)-2, IL-7, IL-10, macrophage inflammatory protein-1A (MIP-1A), and monocyte chemoattractant protein-1 (MCP-1) found in critically ill COVID–19 patients may cause acute respiratory distress syndrome (ARDS), which is the main cause of their death [25–27].

## Serological test kits and vaccines

Current diagnostic tests for SARS-CoV-2 fall into two main categories, namely, the detection of viral RNA via molecular tests and the detection of anti-SARS-CoV-2 immunoglobulins via serological tests [28]. A common molecular test that is applied for viral detection is the reverse transcriptase polymerase chain reaction (RT-PCR) or more commonly known as ‘swab’ tests. However, serological test kits, a blood test, have generated considerable interest as an alternative or as a complement to RT-PCR in the diagnosis of acute infection due to its ability to identify individuals who have contracted COVID-19 despite not undergoing testing while being ill [28]. Many serological test kits have been produced at such a hasty pace that it exceeded rigorous evaluation, leaving uncertainties in its accuracy and effectiveness [29]. Despite the questionable accuracy, some serological test kits might have lower price and easier implementation, such as aUS$ 3 rapid test kit developed by a Bangladeshi lab with results given under 15 minutes, compared to a US$125 ‘gold standard’ RT-PCR test giving highly accurate results with 4 to 6 hours of processing time [30].

Microalgae-derived products may provide some assistance in the production of serological test kits. In particular, test kits that apply immunoassay methods. Since the primary principle of immunoassay testing is the use of an antibody or an antigen that would bind to the antibody of interest, microalgae-derived compounds could be incorporated due to its binding properties. New and innovative ways are being researched by using microalgae to make the necessary proteins (i.e. viral protein antigens) to identify COVID-19 antibodies in victims infected by the disease. Berndt et al. [31] reported that SARS-CoV-2 recombinant receptor-binding domain (RBD) has become the most used antigen for serological tests based on the collective data. Besides acting as a tool to determine antibodies, the recombinant spike protein also plays a role as a vaccine antigen where it is being used to develop the SARS-CoV-2 vaccines [31]. The rise of COVID-19 has incited a vaccine race across the world with the virus taking millions of lives thus far. Research and development initiatives began their journey in developing a vaccine early in the year 2020, once the genetic sequence of SARS-CoV-2 was published [32]. As of 4 March 2021, three COVID-19 vaccines including Pfizer-BioNTech, Moderna and Johnson & Johnson/Janssen have been authorized and recommended by Centers for Disease Control and Prevention, the United States and as of 27 February 2021, clinical testing of COVID vaccines such as AstraZeneca and Novavax have reached phase three trials [33].

Despite recent breakthroughs on the SARS-CoV-2 vaccines, the implementation of microalgae in current research efforts can be considered, as many species are GRAS organisms since human-related pathogens, viruses or endotoxins are absent within them [34]. For example, *Spirulina* has been recognized as GRAS (i.e., safe to consume) since 1981 by the U.S. FDA as it has no risk to human health, whereas *Chlorella* and *Dunaliella* are also considered as GRAS by the FDA [35]. They are unique hosts for the discovery and development of bioactive compounds including different forms of pigments, polyphenols, lectins and polysaccharides, many of which have prophylactic properties [9]. There have been several biopharmaceuticals produced in algae via genetic modification. These include vaccines, some of which have been evaluated at the preclinical trial level such as vaccines against peanut allergies, malaria, and Human papillomavirus [9].

Berndt et al. [31] listed the challenges of non-animal cell production systems for the production of useful virus spike proteins, including bacterial system, which showed poor protein folding, fungal system which had low productivity as well as plant-based systems which had low biomass productivity. However, algae can be scaled up very rapidly, grown photosynthetically or heterotrophically, and demonstrated to fold complicated eukaryotic proteins, to be genetically modified, and to express recombinant proteins [31]. Gene editing technologies have proven its great potential in current advancements as genetic modification ventures into microalgal strains to improve productivity, robustness, harvestability, processability, nutritional composition, and application [36,37].

**Figure 1.**
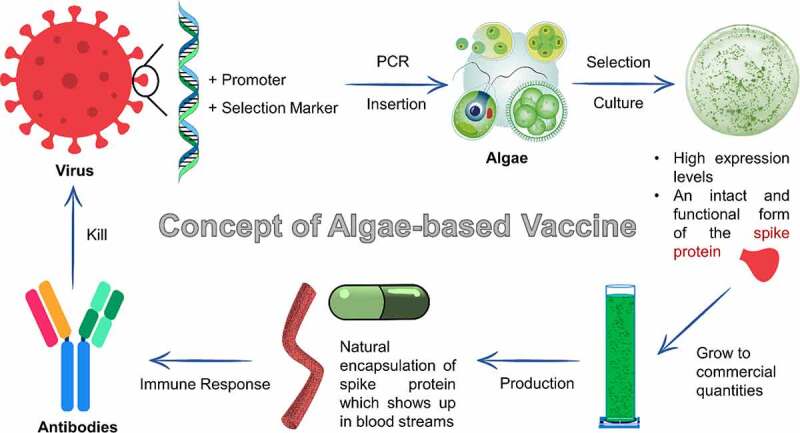
Concept of algae-based vaccine being developed by TransAlgae

Although there are available vaccines for SARS-CoV-2, research efforts on developing algae-based edible vaccines should not be neglected. Basically, the current on-going researches use single-celled alga *C. reinhardtii* where one research group in Italy introduced a DNA sequence corresponding to the SARS-CoV-2 RBD protein by adopting nuclear transgenesis and chloroplast transformation (Table 2). Additionally, Berndt et al. [31] utilized *C. reinhardtii* to produce recombinant SARS-CoV-2 spike RBD protein and found out that it has similar affinity as mammalian expressed in specifically binding to the recombinant ACE2 protein, showing the potential of using algae to produce functional and correctly-folded recombinant spike RBD protein which could be utilized in large-scale serological tests or as potential vaccine antigens. Moreover, a Biotech Company in Israel, TransAlgae identified and inserted a portion of the spike protein into the algae for the manufacturing of spike protein to stimulate immune responses, while claiming that adding the spike protein in tiny amounts does not change the safety profile of the algae for humans [18]. The key feature of using algae for vaccine production is that an oral vaccine can be produced by lyophilizing and encapsulating the algae, where their cell wall could protect the antigens and bioactive molecule from harsh gastric environment, ensuring its arrival to the intestinal immune system [38,39]. Figure 1 demonstrates the concept of algae-based oral vaccine introduced by Gressel [40] in TransAlgae.

**Table 2.** On-going researches on algae-based products being developed for SARS-CoV-2

Product	Country	Target Function	Key Features	Ref.
Algae pill with vaccine inside	Israel	To genetically modify algae to manufacture the spike protein and provide a natural encapsulation	A portion of the spike protein is identified and inserted in algae for subsequent ingestion to generate antibodies and an immune response.	[40]
Algae-based oral vaccine	Italy	To introduce a DNA sequence that encodes an antigen derived from SARS-COV-2 into the microalgae genome	Nuclear transgenesis and chloroplast transformation are used. For each gram of dried algae biomass, up to 1 mg of the recombinant antigen is possible.	[53]
Multipurpose four-layered algae-based respirator (Oxigeno)	India	To filter out harmful gasses and small particles in the air, while increasing the amount of oxygen passing through	Algae cartridge can be changed for reuse purpose and the filters with an inbuilt sensor that will alert the user Made by a biodegradable and antimicrobial plastic called PLA-Active and a silicon membrane which is much gentler on the face.	[47]
Nasal spray (Q-griffithsin using an anti-viral algal protein)	US	To provide an effective level of defense against the coronavirus for immune-compromised individuals who cannot take vaccines	The nasal spray can be utilized for a broad spectrum of diseases, unlike vaccine specific for particular coronavirus. It has been shown to be effective against Ebola, herpes, and hepatitis, as well as SARS and MERS.	[46]
Sulfated polysaccharides from *Porphyridium* sp.	India	These biocompatible compounds can be used as a coating material on the sanitary items for COVID-19 prevention; also for the production of antiviral drugs	Antiviral activity is supported with the immunity boosting property. Selective inhibitors of several enveloped and non-enveloped viruses and act predominantly by inhibiting the binding or internalization of virus into the host cells.	[54]
Heparin and fucoidans from algae	US	To treat coronavirus early and even prevent it	Heparin’s antiviral activity is higher than that of many antiviral drugs, including Remdesivir, which is now actively used in the US to treat coronavirus. Inhaling heparin (e.g., in a spray) has additional benefits such as reducing pulmonary coagulopathy and inflammation without systemic bleeding.	[19]

## Other algal products being developed for Covid-19

Besides serological test kits and vaccines, algae and algae-derived compounds have been suggested to help in other ways against SARS-CoV-2 such as the production of respirator and nasal spray. For example, polysaccharides mentioned in Table 1, or more specifically sulfated polysaccharides from *Porphyridium*, have been utilized in producing antiviral drugs and it is also suggested to be used as a coating or impregnating material on the sanitary items such as tissue paper, facial masks, gloves and cotton swabs [41]. Internalization or binding of virus on the host cells can be inhibited by the exopolysaccharides from *Porphyridium* together with sulfated polysaccharides and carrageenan.

Besides, a recent study by Bansal et al. [42] investigated the antiviral efficacy of different formulations in nasal spray since SARS-CoV-2 initially replicates at rhinopharynx and nasal cavity. It was found that iota-carrageenan inhibits infection of SARS-CoV-2 in Vero cell cultures with concentrations as low as 6 µg/mL. In addition, there was proven viricidal activity by xylitol at a concentration of 5% m/V on its own, besides beneficial association with iota-carrageenan [42]. Interestingly, as an available product on the market, a nasal spray formulated with iota-carrageenan has been shown to be effective and safe against common cold virus [43,44], while reducing mortality of mice infected with lethal doses of H1N1 influenza virus [45]. Moreover, an antiviral protein was found in the New Zealand red algae *Griffithsia*, and used to make nasal spray with objective to give immune-compromised individuals who are unable to take vaccines an effective defense. Effective results against a broad spectrum of coronaviruses, including SARS and MERS, Ebola, herpes, and hepatitis have been shown, while published work is required for confirmation and accuracy  [46].

Apart from that, a research group in India worked on a multipurpose four-layered algae-based respirator that includes HEPA filter to avoid dust particles, activated carbon filter to remove VOCs and odors, nitrogen and sulfur oxide filter to stop harmful air contaminants, PTFE filter to remove particles as small as 0.44 µm, and additional algae cartridge to further enrich the purified air with oxygen [47]. This type of respirator might be useful while its reusability and in-built sensor are a good suggestion for sustainability and practicality. Table 2 summarizes the developing algae-based products up-to-date targeting SARS-CoV-2, to provide insights to the researchers on this kind of potential, innovative and helpful products. However, more intensive and realible studies are required to verify the use and effectiveness of these algae-derived products against SARS-CoV-2, or other viruses, and the associated symptoms. Besides the products under development, it is important to strengthen the immune system since the SARS-CoV-2 is seen fatal for human with weak immunity. *Spirulina* which is highly nutritious with several bioactive compounds and antioxidant, immunostimulant/immunomodulatory and anti-inflammatory effects can be taken as superfood and a natural supplement for stronger immune system [23,48]. Additionally, natural astaxanthin derived from microalgae with antioxidant and anti-inflammatory properties is protective against cytokine storm, acute respiratory distress syndrome and acute lung injury, hence it could be used as adjunctive supplement in combination with primary antiviral compounds, while further validation via clincal studies is required [49]. In brief, algae and algae-derived extracts can be used as the supplement to prevent viral infection or to help during the post-treatment care of the COVID-19 patients, particularly on the side effects associated with drug toxicity.

## Challenges and future perspectives

Algae biotechnology has proven its value in medicinal fields and it presents plenty of potential in contributing to the war with COVID-19. For instance, algae in powders or capsules can be used to improve immunity which could prevent the severe viral infection since prevention is better than cure. Moreover, the properties and features of algae-derived compounds shows great potential in the process of developing serological test kits, vaccines, and other beneficial products. Remarkably, algae have the potential to be genetically modified, dried out and employed directly, omitting out the extraction and purification costs while recombinant antigen which is protected in the algal cell wall for longer periods could be obtained without any efficacy loss, easing the development of vaccine [18]. However, challenges are bound to arise even with this beneficial natural resource.

The current trend toward the commercialization and cultivation of algae is targeted toward nutritional products such as *Spirulina, Chlorella, Dunaliella*, and *Haematococcus* in several dozen small to medium scale production systems around the world. Algal species that are used for the development of pharmaceutical products such as lectin have high production costs [50]. This could prove to be a challenge as further research and development of algae might be unfavorable due to its costly expenses in harvesting. Newer and cheaper methods of harvesting different algal species and strains ought to be developed to allow for an increase in commercialized application for pharmaceutical purposes. However, when comparing to plant-based compounds which also exhibit anti SARS-CoV-2 activity, production cost of algal sulfated polysaccharides is cheaper, owing to their abundant amount in the marine habitat [51]. Additionally, these compounds are soluble in water, easily extractable via an aqueous extraction technique, and easily modifiable while extraction of plant-based compounds requires harmful organic compounds [52]. Nevertheless, chemical composition, bioavailability, biological potency, toxicity, and related mechanisms of algae-derived compounds need to be understood, especially in pharmaceutical sectors.

It can be seen that algae contain huge reservoirs of beneficial elements such as its antiviral properties and protein production. Usage of high-throughput assays to screen valuable algal compounds and selection of the most promising agents will accelerate the discovery of algae-derived anti-SARS-CoV-2 extracts [9]. Current developments in gene editing technologies further adds to the potential of algae contribution toward the efforts to resolve health issues caused by COVID-19. The genetic modification of algae to produce and manufacture the proteins necessary to identify or replicate SARS-CoV-2 proteins should be the main focus in current research efforts. It is also important to enhance the stability of the genetically modified algae strains and the protein yields. Further research in this area has to be done as a form of developing strategies to inhibit the recurrence of viral diseases. Overall, antiviral properties of algae-derived extracts are being researched in vitro but there are still research needs on the practical implications, clinical research, and demonstration scale.

## Conclusion

Research initiatives on SARS-CoV-2 are developing rapidly due to the severe global impact. However, there is yet to be aconcrete cure or solution for this global pandemic. Owing to the urgent need of therapeutics against SARS-CoV-2, it is necessary to search for all potential antiviral agents, especially those with relatively broad-spectrum. This short review presents the remarkable potential of algae and algal bioactive metabolites such as lectins and sulfated polysaccharides since there has been increasing evidence proving their antiviral activities and immunity-boosting capacity. The current developments of several microalgal products such as vaccine and nasal sprays have been summarized to provide further insights into the enormous probability of obtaining novel therapeutic compounds from these natural resources, with the aid of genetic engineering. These products and their roles to help in combating SARS-CoV-2 or other alike viruses warrants urgent developments even though it is too early to conclude the effectiveness of algae against SARS-CoV-2. Hence, intensive efforts are required to verify the use of algal compounds against SARS-CoV-2 and related symptoms, along with the direction of developing effective vaccines and drugs against current and potential future novel infectious viral diseases.
